# Abundance, diversity and domain architecture variability in prokaryotic DNA-binding transcription factors

**DOI:** 10.1371/journal.pone.0195332

**Published:** 2018-04-03

**Authors:** Ernesto Perez-Rueda, Rafael Hernandez-Guerrero, Mario Alberto Martinez-Nuñez, Dagoberto Armenta-Medina, Israel Sanchez, J. Antonio Ibarra

**Affiliations:** 1 Instituto de Investigaciones en Matemáticas Aplicadas y en Sistemas, Universidad Nacional Autónoma de México, Unidad Académica Yucatán, Mérida, Yucatán, México; 2 Departamento de Ingenieria Celular y Biocatálisis, Instituto de Biotecnología, UNAM, Cuernavaca, Morelos, México; 3 Laboratorio de Ecogenómica, Unidad Académica de Ciencias y Tecnología de Yucatán, Facultad de Ciencias, UNAM, Mérida, Yucatán, México; 4 Infotec, Aguascalientes, Aguascalientes, México; 5 Laboratorio de Genética Microbiana, Departamento de Microbiología, Escuela Nacional de Ciencias Biológicas, Instituto Politécnico Nacional, Ciudad de México, México; Institut National de la Recherche Agronomique, FRANCE

## Abstract

Gene regulation at the transcriptional level is a central process in all organisms, and DNA-binding transcription factors, known as TFs, play a fundamental role. This class of proteins usually binds at specific DNA sequences, activating or repressing gene expression. In general, TFs are composed of two domains: the DNA-binding domain (DBD) and an extra domain, which in this work we have named “companion domain” (CD). This latter could be involved in one or more functions such as ligand binding, protein-protein interactions or even with enzymatic activity. In contrast to DBDs, which have been widely characterized both experimentally and bioinformatically, information on the abundance, distribution, variability and possible role of the CDs is scarce. Here, we investigated these issues associated with the domain architectures of TFs in prokaryotic genomes. To this end, 19 families of TFs in 761 non-redundant bacterial and archaeal genomes were evaluated. In this regard we found four main groups based on the abundance and distribution in the analyzed genomes: i) LysR and TetR/AcrR; ii) AraC/XylS, SinR, and others; iii) Lrp, Fis, ArsR, and others; and iv) a group that included only two families, ArgR and BirA. Based on a classification of the organisms according to the life-styles, a major abundance of regulatory families in free-living organisms, in contrast with pathogenic, extremophilic or intracellular organisms, was identified. Finally, the protein architecture diversity associated to the 19 families considering a weight score for domain promiscuity evidenced which regulatory families were characterized by either a large diversity of CDs, here named as “promiscuous” families given the elevated number of variable domains found in those TFs, or a low diversity of CDs. Altogether this information helped us to understand the diversity and distribution of the 19 Prokaryotes TF families. Moreover, initial steps were taken to comprehend the variability of the extra domain in those TFs, which eventually might assist in evolutionary and functional studies.

## Introduction

Regulation of gene expression at the transcriptional level is a fundamental process for maintaining cellular homeostasis. Gene regulation is critical for optimizing proteins and structural RNAs and the subsequent levels of metabolites and other cellular properties [1]. This regulation occurs by sensing changes in the surrounding environment and in the internal cellular state. Transcriptional regulation can happen by multiple means, such as small RNAs and riboswitches, among others, but by far the most common regulatory mechanism involves the action of regulatory proteins, also known as DNA-binding transcription factors (TFs) [2]. In general, TFs are two-domain proteins, with a DNA-binding domain (DBD) in either the amino- or carboxy-terminus, which is involved in specific contacts with the regulatory region of the corresponding cognate genes that result in activation or repression of gene expression. The second domain, which we have named in this article as “Companion Domain” (CD), might have one or many tasks including ligand binding, protein-protein interactions with other proteins (including the transcriptional machinery), and even enzyme activity, or modulating actively the DNA- binding ability of the DBD. In many reports this domain is also called “effector binding domain” (EBD) [3], but it is important to mention that only for a small fraction of these domains a capability to bind effector molecules or proteins has been described and therefore we chose to use our definition. That said, for most of the TFs this accompanying domain or CD has not been extensively studied. Therefore, despite the fact that this CD may have an assignment it is escorting the DBD. Once a TF receives a signal, it modifies its ability to bind specifically to the DNA-binding site and then acts by either activating or repressing gene expression [4].

Therefore, the aim of this project was to evaluate, in a systematic approach, the abundance, diversity and domain architecture variability in 19 families of DNA-binding TFs derived from 761 non-redundant prokaryotic genomes. Our analysis on abundance and distribution of all the identified TFs grouped them in four main classes of families depending on their abundance. In addition, we observed that some TF families have been more evolutionary successful than the others depending on the life style of the organisms. Finally, those TF families with a higher variability in their CDs were identified. This might have some evolutionary implication about which CDs have a higher probability to be found in other TF in the same family. We believe this is an initial step to study and understand the CDs in TFs.

## Materials and methods

### Bacterial and archaeal genomes analyzed

Prokaryotic genomes were downloaded from the NCBI webserver. To achieve a comparative analysis, open reading frames (ORFs) that encode predicted proteins in all organisms were only considered. In addition, redundant genomes were excluded to avoid any bias associated with overrepresentation of organisms as reported by Martinez-Nuñez et al. [5]. In brief, the selection of organisms was carried out through concatenation of 21 conserved proteins across bacteria and archaea genomes. In a posterior step, a single data set for phylogenetic analysis was constructed, and genomes located closely together on a phylogenetic tree were excluded leaving a representative; leaving a set of 761 representative genomes composed by 672 bacteria and 89 archaea.

### Identification of regulatory proteins and companion domains

TFs were identified based on DBD database assignments [6] and two model organisms with experimental evidences collected in RegulonDB v9.0 [7] and DBTBS v5.0 [8] databases. In addition, family-specific hidden Markov model (HMM) profiles were constructed from known TFs and from information in the RegulonDB and DBTBS databases to search for in the bacterial and archaeal genome sequences. The repertoire of TFs identified in the model organisms was cross-checked to calculate the intersection between well-known TFs identified by HMM profiles, i.e. the efficiency of the profiles to identify possible TFs. The HMM profiles were used to identify potential DBDs by setting the E-value at ≤10^−4^ and a coverage of 60%. Those proteins identified by HMM profiles were scrutinized to assess their domain organization by using the Superfamily database assignments [9]. In brief, we used all the library of 1659 superfamily HMM models to run in the HMMer program against the collection of TFs, with an E- value at ≤10^−3^ to be considered as significant.

### Correlations between families and number of different genomes

The abundance of a family in each genome was measured as the number of proteins with at least one predicted hit for the respective family. Diverse families can be present in different proportions in each organism, such as those that occur in only one or two genomes and in only a few proteins or those families widely distributed in all the genomes. For each family, changes in abundance across different bacteria and archaea can be described in an abundance pattern or profile, as previously described reported by Vogel and Chothia [10]. In brief, the abundance counts for one family across different genomes were normalized according to the following formula:

$$A_{n}=\frac{{(A}_{i}-A\_avg)}{A\_sdv}$$

Where *A*_*i*_ and *A*_*n*_ are the absolute and normalized abundance counts, respectively, in a particular genome, and *A*_*avg* and *A*_*sdv* are the average abundance and standard deviations across all genomes for that family, respectively. This means that the abundance of a family in one genome can be described relative to its abundance in other genomes.

### Enrichment analysis

To evaluate the association between a DBD and CDs in the 19 TF families, an enrichment analysis considering a one-tail Fisher’s exact test was conducted, with a statistical significance *p*-value ≤ e−10. Multiple testing corrections were performed using the Benjamini-Hochberg step-up false-discovery rate-controlling procedure to calculate adjusted *p*-values. Rstudio was used to perform all the analyses [11].

### Evaluation of domain architecture diversity

In order to determine the domain architecture diversity associated with TFs, the weighted domain architecture score (WS) was calculated as described in reference [12]. In brief, the method considers the proteins containing a domain and the total number of proteins under study (total proteins per genome) via the inverse abundance frequency (IAF) statistic, as follows:

$$IAF(d)=log2\frac{Pt}{Pd}$$

Where *P*_*t*_ is the number of total proteins (per genome) and *P*_*d*_ is the number of proteins containing domain *d*.

To measure the association of a protein domain, we defined the inverse variability (IV) obtained from the inverse of the number of distinct partner domain families at the N- and C-terminal sides adjacent to a domain, i.e. the diversity of architectures associated to a specific domain. The definition of the IV of a domain, *d*, is:

$$IV(d)=\frac{1}{fd}$$

Where *f*_*d*_ is the number of different domain families adjacent to domain *d*.

Finally, the WS of a domain is the product of the IAF and the IV of a domain:

WS=IAF×IV

## Genome size windows

To evaluate the association between the WS and genome sizes, all the genomes sizes (measured in ORFs) were binned in 11 intervals without overlaps, with a width of 836 ORFs. The number of intervals was calculated by using the Sturges’s formula, which grouped many different values in equal classes: k = 1+ log_2_(N), where k is the number of equal classes and N is the number of data, rounded to the nearest integervalue [11]. Then, the width of classes was determined with the formula: c = R/k, where R is the difference between the high value (small genome) and low value (large genome).

## Results and discussion

### Abundance of regulatory protein families in bacterial and archaeal genomes

In order to determine the abundance of DNA-binding TFs in bacterial and archaeal genomes, 761 non-redundant genomes were evaluated, and TFs belonging to 19 families were identified as described in the methods section [5]. The results summarized in Table 1 and S1 Table show that the most abundant families identified were TetR/AcrR and LysR, which are widely distributed among all the bacterial and archaeal genomes and together represent 28.4% of the complete collection of proteins analyzed in this work. Members of the TetR/AcrR family (15.9 members per genome) are mainly involved in regulation of multidrug resistance, biosynthesis of antibiotics, and pathogenicity (see [13]), i.e. fundamental processes of bacterial resistance. LysR TFs (15.26 members per genome) also regulate a wide variety of transcription units and functions. For instance, in the bacterium *Escherichia coli* K-12 this family is involved in the regulation of amino acid biosynthesis and catabolism, oxidative stress response and detoxification of the cell [7].

**Table 1:** Families of transcription factors identified in the bacteria and archaea genomes.

Family	Total number of TFs (^a^)	Number of different CDs (^b^)	Length size of the CD (± SD)	Enrichment of domains (e−10)	Pearson’s (R) value (^c^)
PhoB	4948 (5.93)	72	136.08 (42.06)	8	0.73
GerE	6074 (7.28)	96	134.32 (43.21)	24	0.71
GntR	6639 (7.95)	76	188.69 (91.35)	9	0.71
MarR	6305 (7.55)*	126	149.56 (72.11)	30	0.69
SinR	5939 (7.11)	133	126.99 (62.05)	35	0.65
ArsR	3314 (3.97)	86	138.97 (56.56)	21	0.65
TetR/AcrR	12097 (14.49)	70	111.91 (25.01)	4	0.65
LysR	11610 (13.91)	24	201.09 (15.79)	1	0.62
AraC/XylS	6860 (8.22)	106	136.78 (52.77)	46	0.61
Crp	2264 (2.71)	53	133.14 (31.75)	4	0.61
Lrp	4119 (4.93)	126	109.55 (51.13)	30	0.60
LexA	611 (0.73)	36	119.99 (31.21)	6	0.53
Iclr	1323 (1.58)	80	164.31 (44.13)	11	0.51
GalR/LacI	3504 (4.20)	25	258.52 (32.84)	1	0.51
Fur	1428 (1.71)	14	84.50 (71.21)	1	0.50
Fis	4143 (4.96)	65	192.78 (53.01)	20	0.47
BirA	1076 (1.29)	58	121.56 (62.46)	9	0.47
ArgR	485 (0.58)	22	84.42 (38.25)	2	0.17
TrmB	746 (0.89)	84	139.84 (69.73)	19	0.12
Total	83485	457 (^d^)		210	

^a^. Numbers in brackets indicate the percentage of proteins corresponding to the total dataset analyzed.

^b^. This number represents the total number of different CDs in each family, not the total number of CDs.

^c^. Correlation value between genome size and proportion of TFs.

^d^. Total number of different CDs identified. Please note that it does not represent the sum of the numbers in this column.

A second group of six families that included AraC/XylS, GntR, MarR, GerE, PhoB, and SinR families, with an average of 8.01 members per genome, represents 44% of the total of proteins identified in this work. These families regulate a large diversity of functions, such as carbon source assimilation, multiple antibiotics responses, and phosphate regulation, among others [14, 15]. A third group of families with an average of 4.12 TFs per genome was identified and included Lrp, Fis, GalR/LacI, and ArsR (representing the 18% of the total of the proteins evaluated). Finally, in a fourth group, seven families with a small number of members (an average of 1.19 members per genome) were identified and grouped together, representing a total of 9.5% of the collection, including ArgR, Fur, LexA, IclR, Crp, BirA, and TrmB. Indeed, protein members of these families are present usually in only one copy per organism or are even absent. These results suggest that, based in their abundance, some of the families described above have been evolutionary more successful in bacteria and archaea; whereas small families have been less successful in terms of the number of members per genome. However, they could be associated with global regulation, as it has been described for Crp in *Escherichia coli* K-12, for which two family members have been identified and almost 25% of their genes are under the regulation of this global regulator in some organisms [16].

### Probable expansions as a consequence of duplication events

In order to evaluate how genome size has influenced the repertoire of TFs, we relied on the number of ORFs per organism, under the hypothesis that organisms with a large number of ORFs could be associated with more duplication events, whereas organisms with small genomes would be associated with a minor number of duplication events [5, 17]. To this end, we normalized the abundance of each family of TFs per genome, as described in the Methods section, and an abundance profile was displayed. Posteriorly, for each family, we calculated the Pearson correlation between the abundance profile and the estimated number of ORFs (Table 1). From this, 3 families (GntR, GerE, and PhoB) showed a strong correlation between their abundance and the number of ORFs, with an R-value of ≥ 0.70. This result suggests an expansion of these families in almost all bacteria and archaea, with intermediate abundance in organisms with genome sizes between 1000 and 2000 ORFs, and low frequency or even absent in organisms with small genome sizes (less than 1000 ORFs). Interestingly, these families were included in the second group of abundant families described in the previous paragraph. Therefore, their duplication events could be associated preferentially with an increase in genome sizes. Twelve families, including LysR, TetR/AcrR, and GalR/LacI, have a correlation coefficient (R) between 0.47 and 0.69. These families are substantially increased in some genomes, but this is not directly related to the increasing of genome sizes. Finally, two families (TrmB and ArgR) have correlation coefficients of less than 0.2, suggesting that these families are in low copy number or even absent in bacteria and archaea genomes. Probably, these families have been replaced with alternative regulatory processes in diverse organisms; it also suggests that their absence does not compromise the response of bacteria and archaea to diverse stimuli.

In summary, we consider that the GntR, GerE, and PhoB families follow a similar trend of duplication and loss events as a function of genome dynamics, i.e. when the genome is duplicated, members of these families are also duplicated, but when gene loss occurs, these families are affected, increasing or contracting the family, respectively. In contrast, highly abundant families, such as LysR and TetR/AcrR, do not follow this trend, i.e. these families are abundant in some organisms (i.e. 250 members of the TetR/AcrR family in the bacterium *Amycolatopsis mediterranei* U32) or scarce in organisms with the same genome size (i.e. 46 members of the TetR/AcrR family in the bacterium *Sorangium cellulosum* 56); both genomes are of similar sizes (around 9200 proteins). These data reinforce the scenario proposed by Itzkovitz et al. [13] for the evolution of DBDs, where some organisms preferentially use some DBDs and when these DBDs reach the corresponding upper bound, new DBDs are needed. At such points, organisms shift their TF usage to novel TF families or families with fewer members but with more degrees of freedom and higher maximal numbers.

### Lifestyle influences the content of TF families

To determine the influence of lifestyle on the content of TF families, organisms were grouped into four classes according to their lifestyle, in accordance with previous reports [18, 19] and expanded with information provided in the corresponding literature deposited in the NCBI database [20] as well as in the BacMap Genome Atlas [21]. Taking in consideration this classification free-living organisms included 368 genomes, considering nonpathogenic bacteria, even when these organism have the ability to act as symbionts with plants or animals; pathogens (187 genomes), includes organisms reported to produce illness in plants or animals despite the fact that some have stages in their life cycle where they survive as free-living organisms; extremophiles (158 organisms), those living in extreme environmental conditions; and intracellular pathogens (48 organisms), which includes obligate endosymbionts and intracellular pathogens.

**Fig 1:**
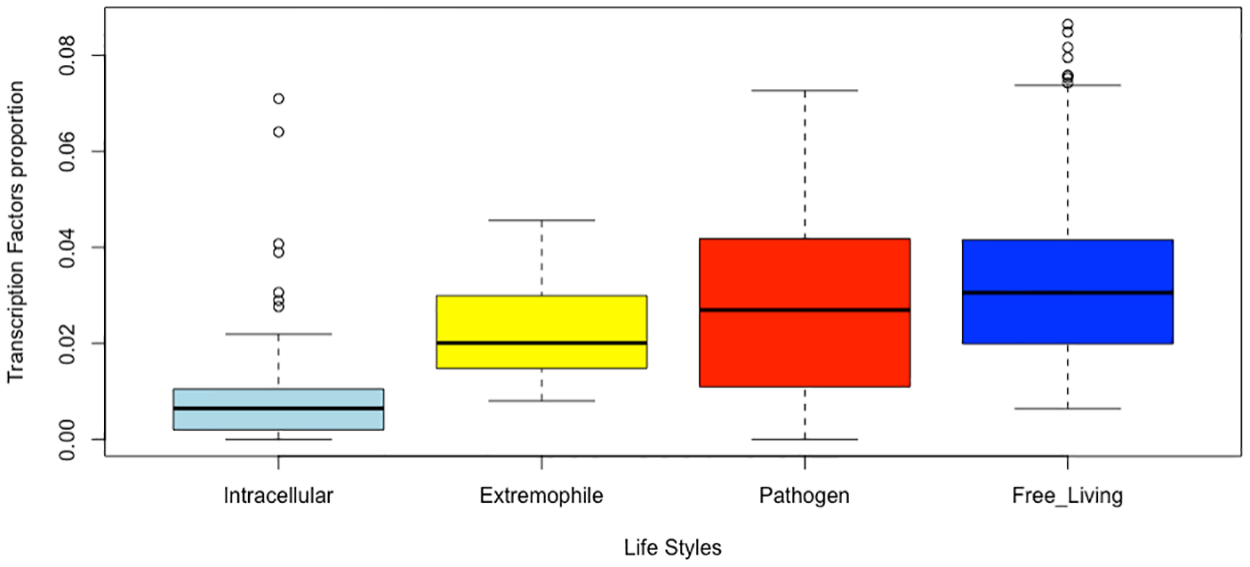
Proportion of TFs in organisms depending on their lifestyles. Organisms were classified in either of four categories: intracellular, pathogens, extremophiles, and free-living, in agreement with [18]. TF proportions were calculated as the ratio between the total number of TFs and the genome size (in ORFs). The line shown inside the box is the median value. The whisker caps represent the minimum and maximum values. Points outside the bars represent the outlier genomes.

This analysis is relevant under the hypothesis that organisms with similar lifestyles would have a similar repertoire of TFs, because TF family abundance is influenced by the environment an organism inhabits. Therefore, we plotted the proportion of TFs per genome according to lifestyle (Fig 1). Based on this analysis, we found that organisms in the free-living category, with their larger genomes, had a higher proportion of TFs (Kruskal-Wallis test *P*-value, <2.2e−16), while intracellular organisms exhibited the lowest TF content, which correlates with their small genomes. On the basis of these data, we suggest that the fluctuating environmental conditions encountered by free-living bacteria favor increases in TF content, but this is also associated with the genome size, as previously reported [19]. These results reinforce the idea that free-living organisms have a greater percentage of TF-encoding genes, while intracellular organisms have a lower proportion of genes devoted to gene regulation.

**Fig 2:**
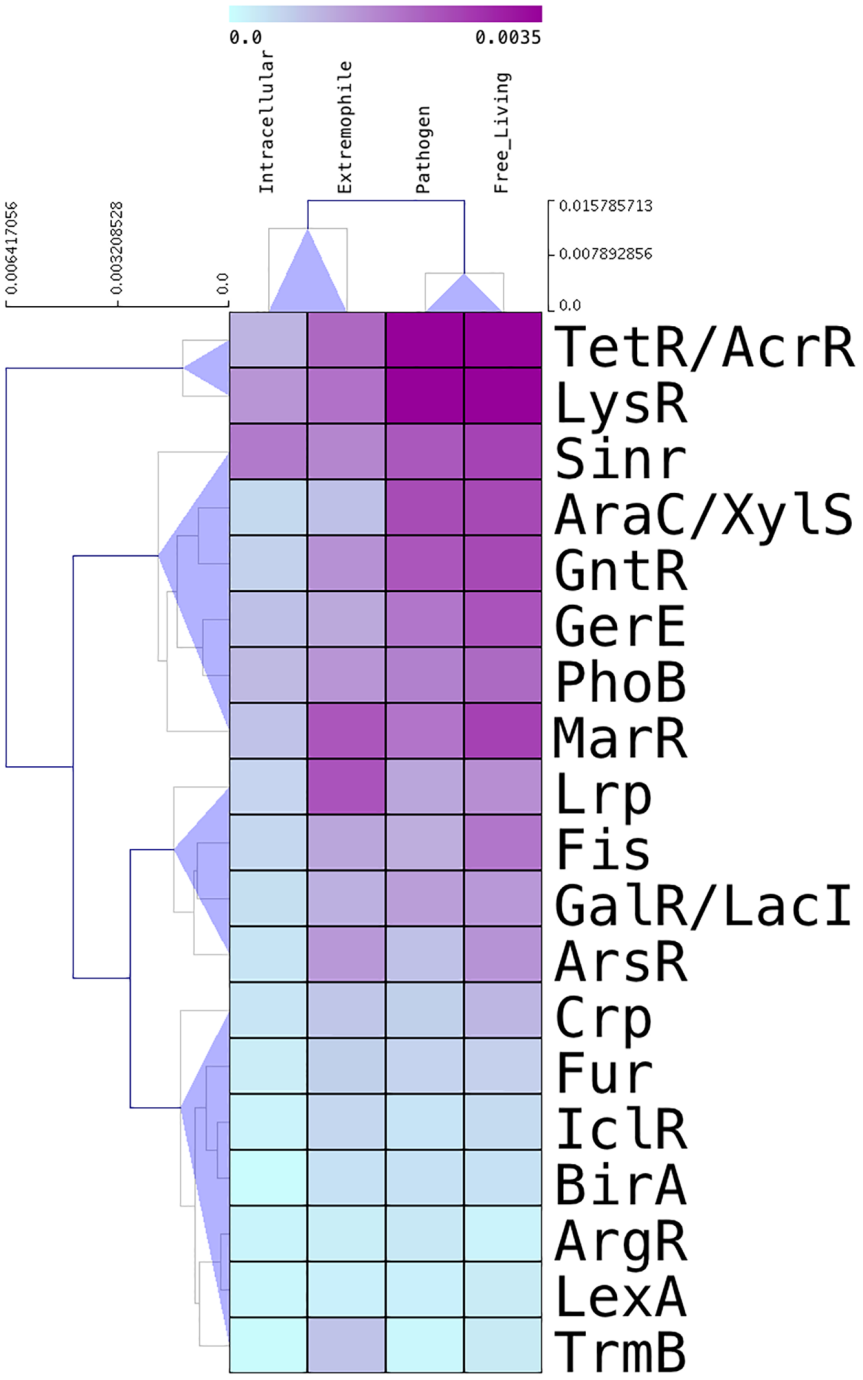
Distribution of TF families per lifestyle group. Each column denotes a life style as described in Fig 1, whereas rows denote the 19 TF families analyzed in this work. The heat map bar at the top of the figure indicates the relative abundance of family per lifestyle. Four groups of TF families were identified based in a hierarchical clustering approach by using a Manhattan distance and a supporting tree with an average linkage algorithm, also with a correlation uncentered as a similarity measure. Numbers on top of the TF families denote the proportion of these TFs and the numbers in the upper left section show the weight scores (WS).

To expand these observations, we decided to explore the proportion of families according to lifestyle, under the hypothesis that large families will contribute significantly to the repertoire of TFs in all bacteria and archaea. To do this, we calculated the rate of occurrence of each family for the number of ORFs per organisms and per lifestyle, and a hierarchical clustering approach with a Manhattan distance and support tree with average linkage algorithm, with correlation uncentered as a similarity measure, was implemented in the Mev4 program [22]. In this regard, Fig 2 shows that LysR and TetR/AcrR families contribute significantly to the total repertoire of TFs in organisms of all of the lifestyles, i.e. they are ubiquitously distributed in all the organisms. In contrast, the SinR, AraC/XylS, GntR, GerE, PhoB, and MarR families contribute to the repertoire of TFs in extremophilic, pathogenic, and free-living organisms; whereas the Lrp, Fis, GalR/LacI and ArsR families contributed to the extremophilic TFs and free-living organisms. Finally, the TrmB, Crp, Fur, IclR, BirA, ArgR, and LexA families does not have an evident contribution to the repertoire of TFs in the organisms as a function of lifestyles, except for TrmB that contributes to the repertoire of TFs of the extremophile organisms. Taken together these observations suggest how each family is distributed in each group of organisms and also that perhaps the environmental pressure (or the absence of it) might affect the abundance/scarcity of each TF family.

### Protein architecture diversity in the transcriptional factors

In order to gain insights into the protein architecture of the 19 TFs families, the complete set of regulatory proteins was analyzed in terms of the composition of structural domains with special emphasis in the extra domains, or CDs. From this analysis, we found that 32% of the TFs are monodomain proteins (or monolythic), almost 59% of the TFs are organized in two domains, and the remaining 9% exhibits three or more structural domains. In a posterior step, all these proteins were evaluated according to their WS that considers the diversity of association between the DBD and the companion domains, even when the DBD is only present. This information was useful to determine how diverse the CDs are within these families. As mentioned in the Introduction section, a CD was defined as a structural domain in the same polypeptide but not forming part of the DBD. Consequently, a DBD might not have a CD at all or it might have one or several CDs. Therefore, from this structural dissection, we found a total of 457 different domains associated with all DBDs in the TF collection (Table 1 and S1 Dataset). These domains were classified into 239 different superfamilies and they can be functionally associated to 35 functional categories according to the Superfamily Database, being the metabolism, regulation and signal transduction the most significant functions (S1 Fig). From this, the most abundant CDs were associated with the most abundant families, such as the LysR, which contains 24 different CDs, mainly associated to the periplasmic-binding protein-like II (PBP II), followed by TetR/AcrR, which contains 70 different CDs (Table 1), being the most abundant domain the associated to tetracyclin binding at the C-terminal domain; and AraC/XylS with 106 different CDs, where the most abundant domain is associated to the arabinose binding and dimerisation domain. Thus, CDs are distributed in different proportions among the TF families and their association with the DBD could contribute to respond to different stimuli.

**Fig 3:**
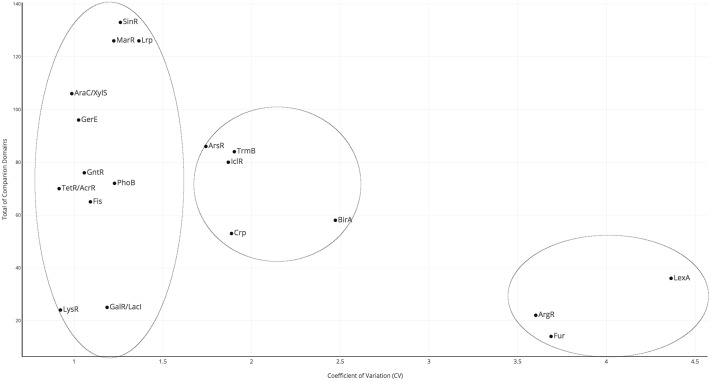
Coefficient of Variation per family. In order to define how complex where the TF in the multiple families TF families were grouped into three classes (indicated by circles) depending on their coefficient of variation (CV) as follows: 0.9–1.36, highly promiscuous; 1.74–2.5, intermediate promiscuity; 3.5–4.5, not promiscuous or monolithic. On the X-axis the CV per family is indicated. On the Y-axis the total of Companion Domains is indicated. CV was determined as the ratio of the standard deviation to the mean for all the WS in the corresponding TF family (for more details please refer to Methods sections).

Therefore, protein architectures associated with all these TF families were evaluated, according to the formula described in reference [12]. In a posterior step, the weight score (WS) as a measure of domain promiscuity in non-redundant proteomes of bacteria and archaea was determined and plotted as a function of the genome size. Obtained results were interpreted as follows, values closer to 0 represent promiscuity and higher values suggest no diversity at all in the protein architecture and, in consequence, proteins must be considered as not promiscuous or monolithic (See S2 Table). In a posterior step, to identify similar groups of families based the WS per genome we calculate a coefficient of variation and, three groups were identified: highly promiscuous families (CV between 0.99 and 1.36), slightly promiscuous families (CV between 1.74 and 2.47), and monolithic families (CV between 3.6 and 4.3) (Fig 3).

**Fig 4:**
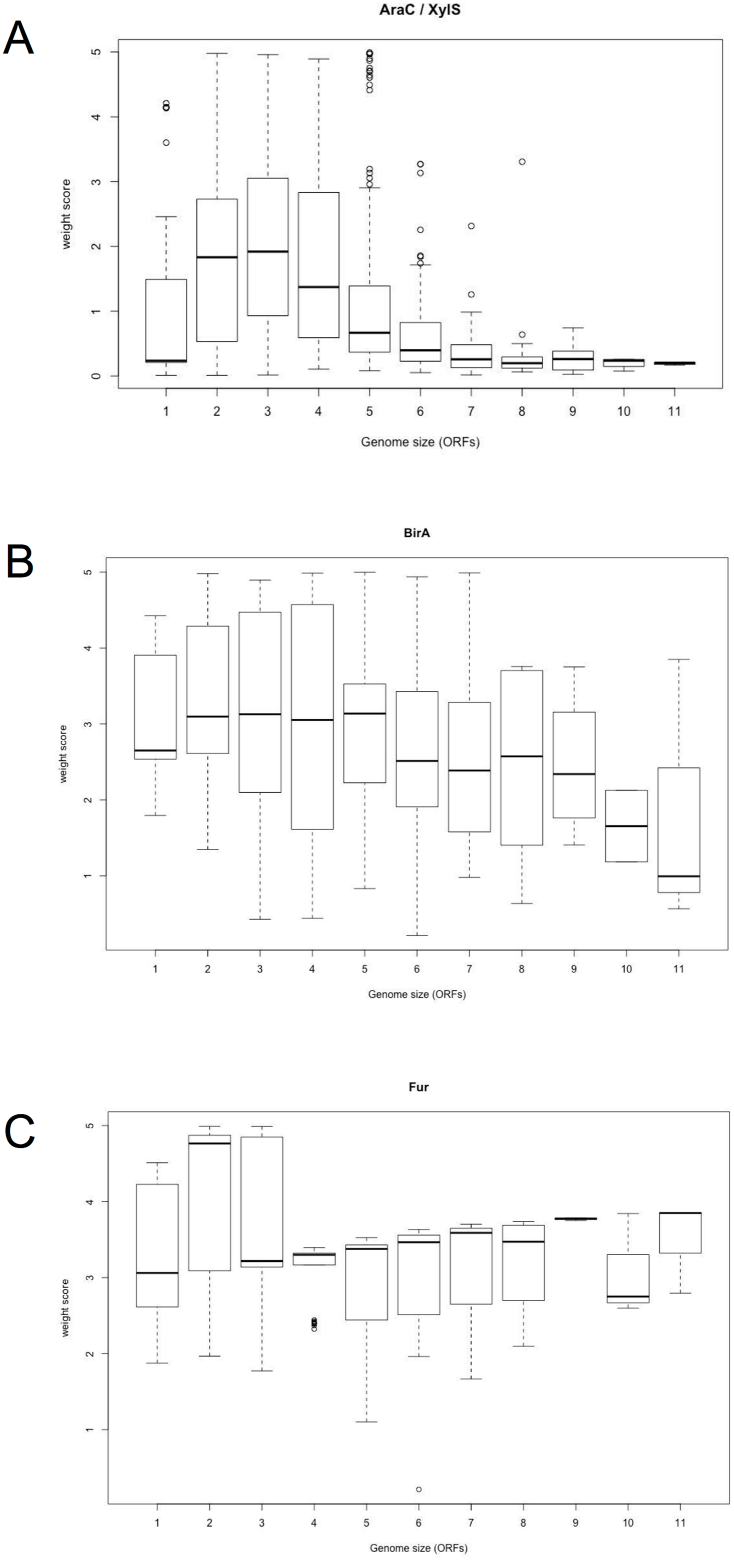
Architecture of families as a function of the genome size. Analysis and classification of the multiple TFs classified them in 3 groups as shown in S1 Table and Fig 3. Examples of these three groups are shown as follows: a) highly promiscuous, the AraC/XylS family; b) intermediate promiscuity, the BirA family; and c) not promiscuous or monolithic, the Fur family. On the X-axis of each graph the genome sizes are displayed in eleven windows with a length of 836 ORFs each (See Methods). On the Y-axis, the weight score (WS) is represented (see the Methods section for details). The line shown in the box is the median value. The whisker caps represent the minimum and maximum values.

In the first group were included the highly promiscuous families of LysR,TetR/AcrR, AraC/XylS, GerE, GalR/LacI, MarR, PhoB, Fis, GntR, SinR and Lrp, with WS values closer to 0 than the other families (Figs 3 and 4a and S2 Fig). The promiscuity index value observed correlates with the number of CDs shown in Table 1 and increase in relation to genome size. In addition, when an enrichment statistical analysis (relationships of less than e−10 were considered significant) was achieved to determine whether there is a significant relation between the DBD and the CD, we found a high number of enriched and unique CDs per family, suggesting an increase in their ability to sense a wide diversity of compounds. LysR family includes the most common TFs found in nature [23, 24], and their CDs are also very diverse. In particular, the AraC/XylS family of transcriptional regulators includes diverse proteins that control the expression of genes involved in diverse biological processes, such as metabolism of carbon sources, pathogenesis, and stress responses, among others [15, 25]. These proteins usually contain a DBD and a CD that are involved in effector/multimerization function. The DBD is a conserved region of around 60 amino acids that contains two helix-turn-helix (HTH) DNA-binding motifs separated by one α-helix [15, 25]. In contrast, the CDs exhibit a large diversity of length sizes (an average of 136.7 ± 52.7 amino acids) and for several members of this family it has been shown to sense environmental signals by interacting with them. For instance XylS and BenR are able to detect the presence of aromatic compounds such as toluene [15, 25, 26]; AraC, RhaS and MelR are able to detect sugars; RegA from *Citrobacter rodentioum* detects bicarbonate; TxtR from *Streptomyces scabies*, cellobiose; UreR form *Proteus mirabilis*, urea; ToxT (*Vibrio cholerae*), bile salts; InvF (*Salmonella enterica*) and ExsA (*Pseudomonas aeruginosa*) bind to another protein (SicA and ExsD, respectively) to exert their role [15, 25, 26]. All these examples are a clear support for our point about this family being one of the most “promiscuous” having CDs with a wide possibility of interactions. In our analysis, the 46 identified CDs were not shared with other families and therefore were unique to this family. Another interesting case represents the GntR family included in this group of promiscuous families that correlates with previous analysis describing the division of this family into four subgroups, according to the type of C-terminal CD (FadR, HutC, MocR, and YtrA) and two minor subfamilies (AraR and PlmA). The C-terminal effector-binding and oligomerization (E-O) domain imposes steric constraints on the DBD, influences the HTH motif and plays an important role in regulation [27]. For example, the E-O domain is able to restrict DBD flexibility in the GntR family, reducing its ability to adapt to varying distances between the parts of a palindromic motif, which reflects on the binding motif structure and therefore in its interaction, or not, with the regulatory region [28].

The second group included those families defined as slightly promiscuous, because their weight scores are slightly related to the genome sizes, such as BirA, Crp, TrmB, IclR, and ArsR (Fig 4 and S2 Fig). These families contain between 53 and 86 different CDs, and their association seems to be similar in bacterial and archaeal genomes. The enrichment analysis identified between 4 and 21 specific domains (Table 1). In this second group are included some moonlighting proteins, such as BirA, where the N-terminal domain is required for both transcriptional regulation of biotin synthesis and biotin protein ligase activity. The role of the wing associated with the DBD in the BirA enzymatic reaction is to orient the active site and protect biotinoyl-5′-AMP from attack by solvents [29]. Global regulators (Crp) were also identified with low diversity of CDs, where the cAMP domain is the representative. These results showed that the combination of significant structural domains identified in all the families provides diversity that affords abilities to sense a wide diversity of environmental signals.

The third group included the monolithic families, such as ArgR, Fur, and LexA (Fig 4 and S2 Fig). These families contained high weight scores independent of the genome size, suggesting low diversity in their protein architectures among the organisms analyzed. This finding correlates with the fact that these families contain a low proportion of diverse CDs, including specific ones. Such as the LexA family of transcriptional factors, usually composed of a DBD and a CD, which is mostly involved in proteolytic cleavage [30]; this same domain organization was a constant protein architecture in all the organisms analyzed for this type of proteins. The Fur family, which is primarily associated with the “Fur C-terminal domain,” comprises members with a common structure that are mainly involved in iron and zinc metabolism (a few are involved in the peroxide stress response) [31]. Therefore, members of this family also show some promiscuity. Finally, the DBD that defines the ArgR family is the “arginine repressor C-terminal domain,” and it is the same in almost all the ArgR-related proteins in bacterial and archaeal genomes [32]. Despite the fact that identity is 27% among the members of this family, it has been shown that they are involved in metabolism and have structural similarity (reviewed in reference [32]).

**Fig 5:**
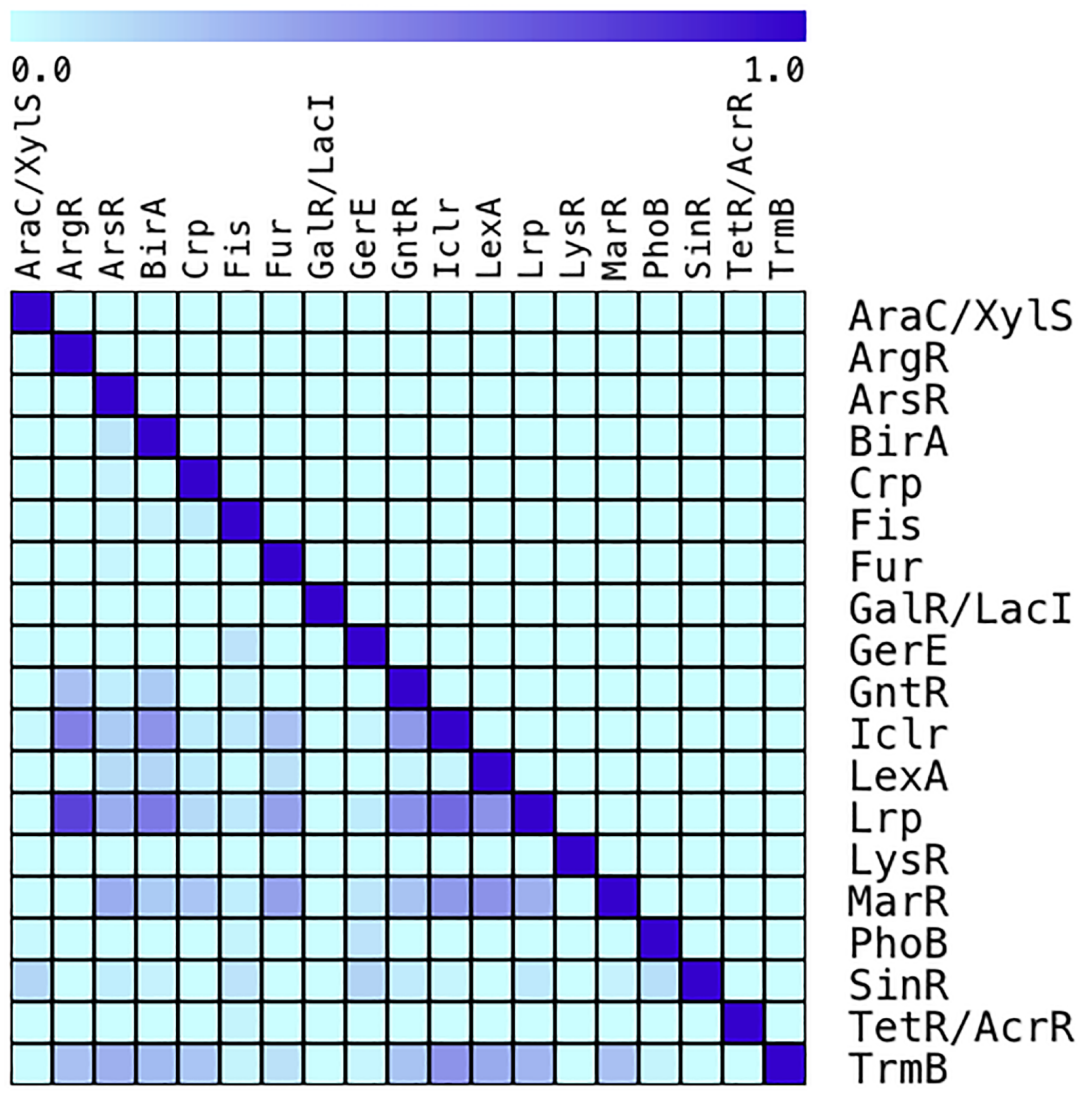
Proportion of CDs shared between different families. In order to identify companion domains (CDs) that are common to more than one family of TFs each of the CDs associated to TF families was compared against the other families. Only domains that were identified as enriched were plotted as a heat map (upper section), in which 0 represents absence and 1.0 represents 100% of CDs in common with two or more families.

Finally, we asked how the CDs are distributed among the TF families, to identify CDs associated to more than one family and those CDs specifically associated to some families. Fig 5 shows which of the tested families had higher promiscuity, but this time the level of promiscuity was based on sharing the CDs with other families. From this analysis, it is clear that the ArsR, IclR, Lrp, MarR, and TrmB families share many CDs with other families, while other families, such as Crp, GalR, Fur, BirA, ArgR, AraC/XylS, Fis, and PhoB, share fewer CDs. The fact that a CD is shared by many TF families might be explained by domain shuffling among proteins, in which gene fusion plays a central role [33]. If this is true, then those families with a high sharing rate are the most promiscuous among this type of proteins, while those not sharing beyond the family can be considered “conservative”. This would mean, in another words, that those in the former group might be subjected to more domains shuffling than those in the latter. Further analysis, searching for this would be interesting to perform.

In summary, characterizing and analyzing CDs not only gives a hint about their distribution, variability and function, but also on how they evolved and are shared among TFs. These domains (also referred as EDBs) might be of significance in the synthetic biology field, this because the more we know about their function and origins the more we can use them as “biological detectors” [3].

## Conclusions

In this work, we have evaluated 19 families of DNA-binding TFs for their architectures in terms of structural domains, how abundant they are in the bacteria and archaea genomes, how they are distributed according to organism lifestyle. Moreover, an insight on how these CDs are shared within and between the families is also discussed. For the examined families, an abundance degree was defined, suggesting which of them have been evolutionarily successful. As for lifestyle, those that are free-living have the most prosperous families, which also happen to be the ones with the highest promiscuity. This combination could be explained because precisely these types of organisms are in contact with the environment and other organisms, which can transfer and acquire genetic material. Our data reinforce the notion that increased gene complexity also requires the development of mechanisms for gene regulation at the transcription level [34], in particular, the combination of DBDs and CDs, suggesting that the interplay of these structural domains could increase the ability of the organisms to recognize and respond to diverse environmental stimuli. Also, a bias towards specific associations between DBDs and CD was identified that depended on the CDs present and the frequency at which the TFs were grouped into the three classes, based on the promiscuity of the CDs. In summary, in this study we found that the protein architectures, duplication events, and the interplay of them in association with genome sizes could help organisms contend with gene complexity at the transcriptional level, increasing the ability of bacteria and archaea to recognize and respond to diverse stimuli and environment challenges.

## Supporting information

S1 Fig Functional categories of superfamily associated to CDs. 239 superfamilies were classified into one of the 6 major categories (General, Information, Intra-cellular processes, Metabolism, Regulation, and other). On the X-axis is the family name. On the Y-axis is the proportion of functional category.
(TIFF)

S2 Fig Box plot of weight scores for the TF families. Analysis is shown for member of the highly promiscuous group: A) TetR/AcrR, LysR, GerE, GntR, Fis, GalR/LacI, MarR, PhoB, and SinR, Lrp; B) intermediately promiscuous: ArsR, IclR, Crp, and TrmB; and C) monolithic or non-promiscuous: ArgR, Fur and LexA. On the X-axis, the genome sizes are displayed in eleven windows with a length of 836 ORFs. On the Y-axis, the WS is represented. The mean of each window is displayed with a line. TF families were grouped into three classes depending on their CV, as follows: 0.9–1.36, highly promiscuous; 1.74–2.5, intermediate promiscuity; 3.5–4.5, not promiscuous.
(PDF)

S1 Table Total of TFs per genome. Columns are as follows: First column indicates the life style as described in the Methods section; second column shows the organisms name of the genome analyzed; columns 3 to 21 indicate the TF families and the number of members per family identified; finally, column 22 shows the total TFs for each organism.
(XLSX)

S2 Table Weight scores associated to the families per genome. Weight scores (WS) were calculated as described in the Methods section. Columns are as follows: column 1, name of the organism; column 2, number of identified TFs for each organism; column 3 to 21, weigh scores for each TFs family.
(XLSX)

S1 Dataset Transcription factors identified in bacteria and archaea genomes. The information of genomes and transcription factors is organized in a tabular format. The file S1_Dataset.zip contains 761 files, one per genome. The information is organized as follows: Genome ID, identifier from NCBI database; Supfam ID; physical position of the Domain; E-value; Supfam model assignment; Supfam description; E-value associated to the family; Family ID; Family ID; representative PDB associated to each assignment.
(ZIP)
