# Supplementary figures and images for: Abundance, diversity and domain architecture variability in prokaryotic DNA-binding transcription factors
Source: PLoS One. 2018 Apr 3;13(4):e0195332. doi: 10.1371/journal.pone.0195332 (PMC5882156; doi:10.1371/journal.pone.0195332)

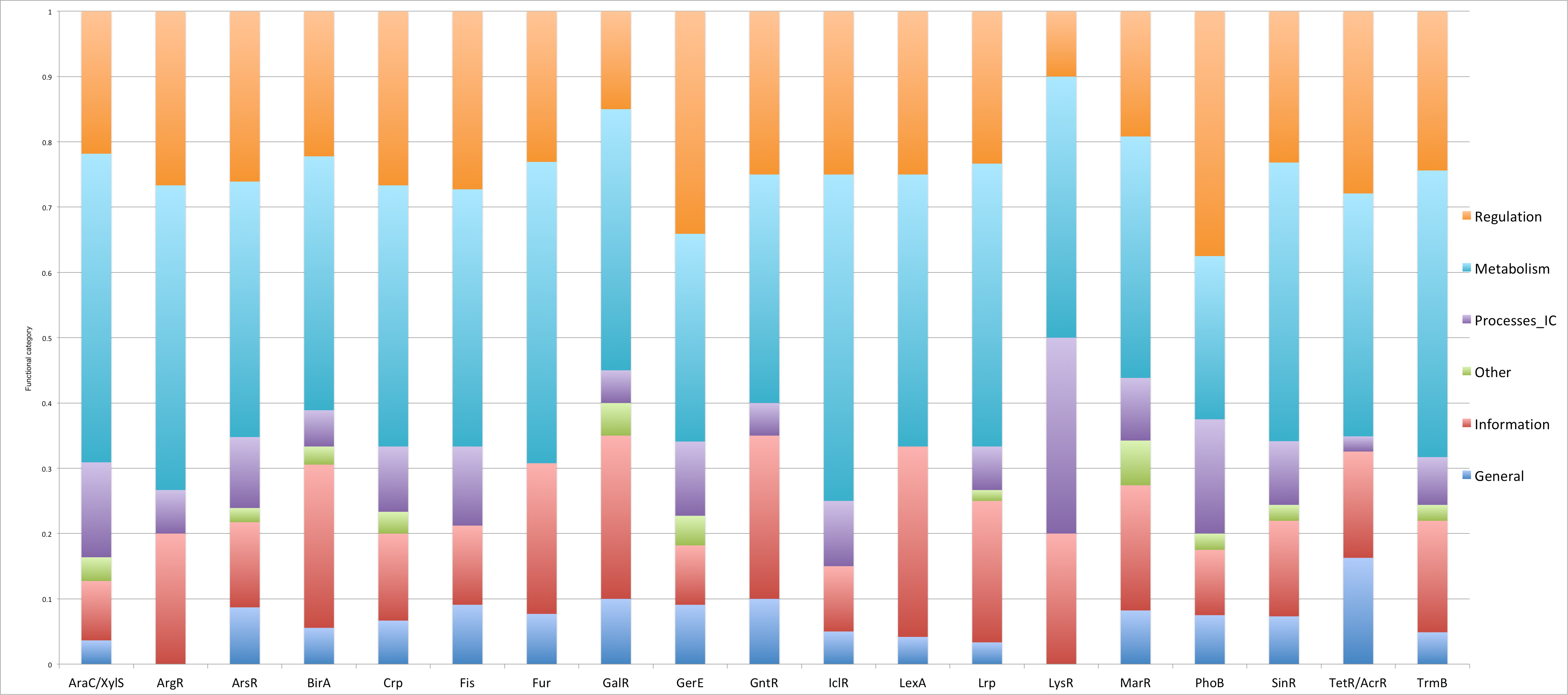

Supplement: S1 Fig — 239 superfamilies were classified into one of the 6 major categories (General, Information, Intra-cellular processes, Metabolism, Regulation, and other). On the X-axis is the family name. On the Y-axis is the proportion of functional category. (TIFF) [file pone.0195332.s001.tiff]

Fig. S2

A

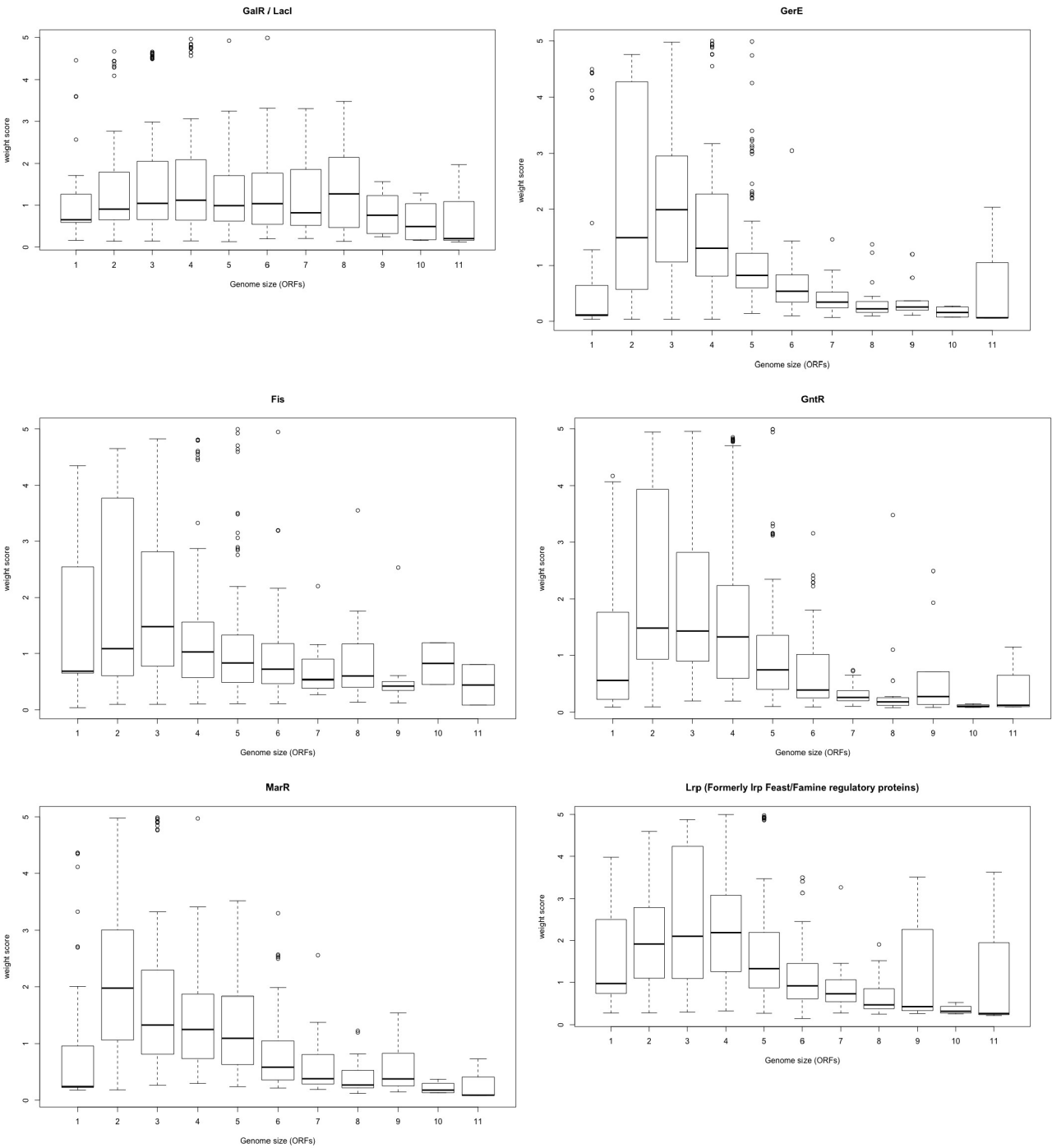

A cont.

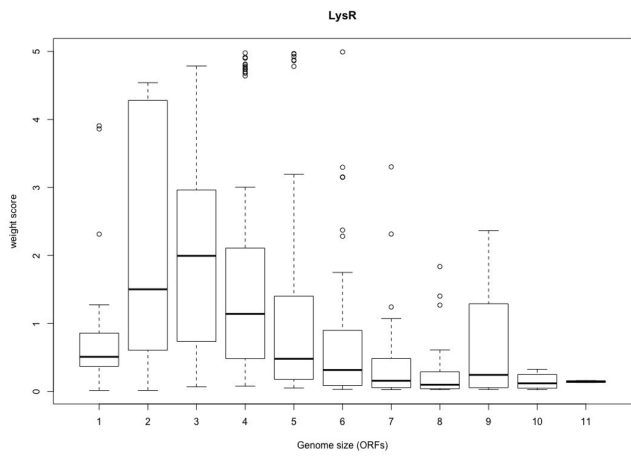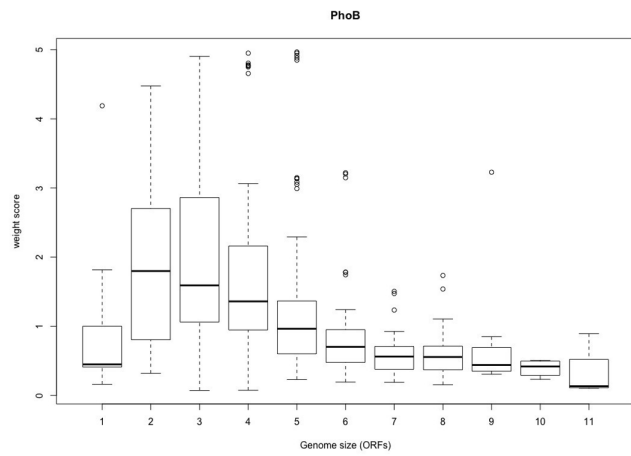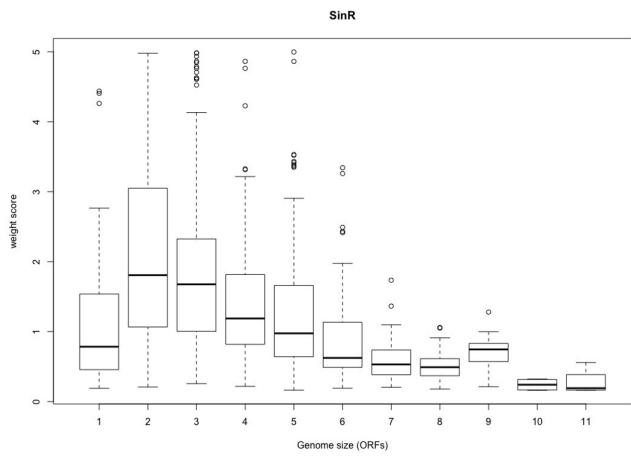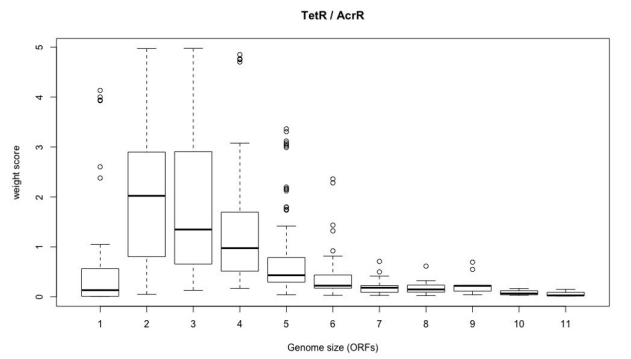

Fig. S2

B

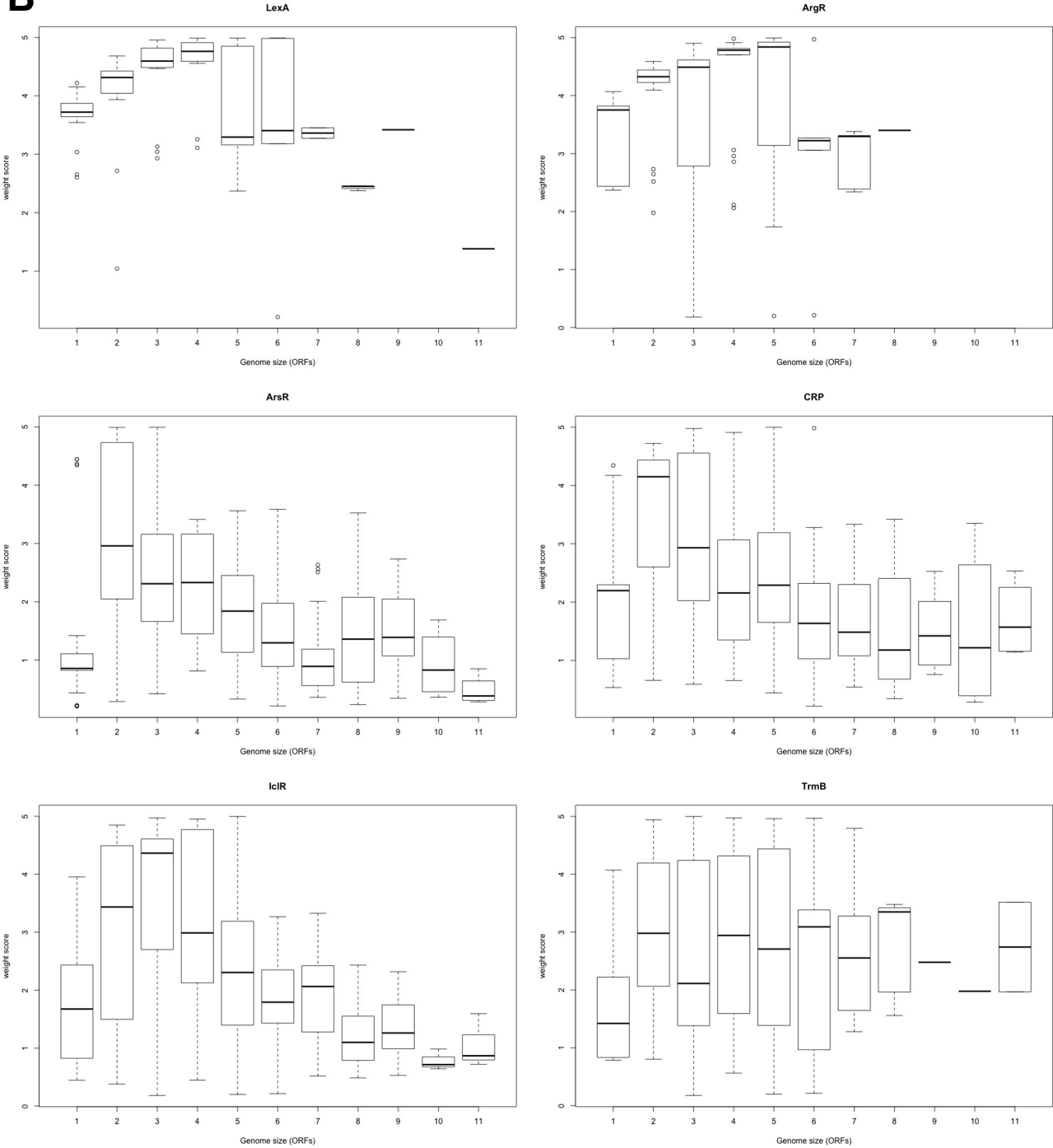

Fig. S2

C

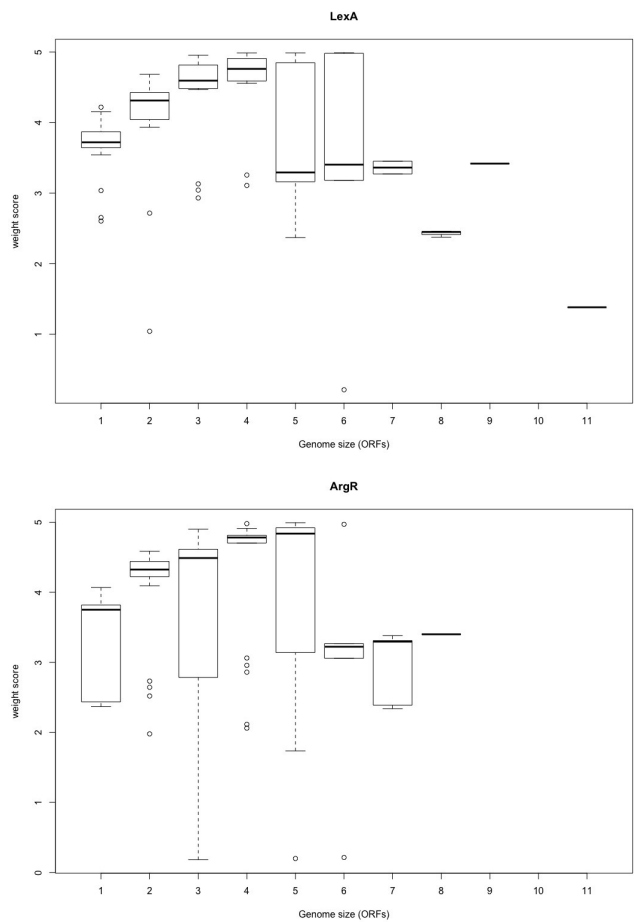

Supplement: S2 Fig — Analysis is shown for member of the highly promiscuous group: A) TetR/AcrR, LysR, GerE, GntR, Fis, GalR/LacI, MarR, PhoB, and SinR, Lrp; B) intermediately promiscuous: ArsR, IclR, Crp, and TrmB; and C) monolithic or non-promiscuous: ArgR, Fur and LexA. On the X-axis, the genome sizes are displayed in eleven windows with a length of 836 ORFs. On the Y-axis, the WS is represented. The mean of each window is displayed with a line. TF families were grouped into three classes depending on their CV, as follows: 0.9–1.36, highly promiscuous; 1.74–2.5, intermediate promiscuity; 3.5–4.5, not promiscuous. (PDF) [file pone.0195332.s002.pdf]
